# Optically Active Nanomaterials and Its Biosensing Applications—A Review

**DOI:** 10.3390/bios13010085

**Published:** 2023-01-04

**Authors:** Santosh Kumar, Zhi Wang, Wen Zhang, Xuecheng Liu, Muyang Li, Guoru Li, Bingyuan Zhang, Ragini Singh

**Affiliations:** 1 Shandong Key Laboratory of Optical Communication Science and Technology, School of Physics Science and Information Technology, Liaocheng University, Liaocheng 252059, China; 2 College of Agronomy, Liaocheng University, Liaocheng 252059, China

**Keywords:** optically active nanomaterials, carbon-based nanomaterials, inorganic-based nanomaterials, organic-based nanomaterials, composite-based nanomaterials, biosensing applications

## Abstract

This article discusses optically active nanomaterials and their optical biosensing applications. In addition to enhancing their sensitivity, these nanomaterials also increase their biocompatibility. For this reason, nanomaterials, particularly those based on their chemical compositions, such as carbon-based nanomaterials, inorganic-based nanomaterials, organic-based nanomaterials, and composite-based nanomaterials for biosensing applications are investigated thoroughly. These nanomaterials are used extensively in the field of fiber optic biosensing to improve response time, detection limit, and nature of specificity. Consequently, this article describes contemporary and application-based research that will be of great use to researchers in the nanomaterial-based optical sensing field. The difficulties encountered during the synthesis, characterization, and application of nanomaterials are also enumerated, and their future prospects are outlined for the reader’s benefit.

## 1. Introduction

Sensor technology, communication technology and computer technology are known as the three pillars of the modern information industry. Nowadays, the research and development of sensor technology has attracted great attention, especially the new sensor technology based on biological principles. The history of biosensors can be traced back to 1962, when Clark et al. [1] creatively proposed the glucose biosensor prototype, that was based on the electrochemical catalytic mechanism of oxygen molecules as electron carriers for indirectly measuring the glucose content in solution. In 1984, Cass et al. [2] from Oxford University reported the fabrication of amperometric glucose biosensor, the ferrocene was employed as electron acceptor in enzyme redox process. This work marks the birth of the second generation of medium enzyme biosensor and opens up a path for the vigorous development of biosensor fields. The third generation of biosensors is the introduction of nanomaterials into biosensors to improve detection sensitivity and broaden the application scenarios. The nanomaterials based-biosensors own unique chemical and physical properties such as surface effect, micro size effect, quantum effect and macro quantum tunneling effect, showing unique superior performance [3]. Modern biosensors are developed on the basis of various signal transmission principles (such as fluorescence signal, refractive index (RI), conductivity and so on) to achieve a clear and simple detection of various objects to be measured. At present, biosensors have shown strong application potential in basic research, biomedicine, environmental and food quality monitoring and other fields [4,5,6].

The mutual integration and collaborative development between the subjects have become the trend of science in recent years. Therefore, the combination of material science and sensing technology has a broad prospect and very important research significance. In addition, many applications require the characteristics of sensors such as small size, integration, low cost and portability [7]. Biosensors based on optically active nanomaterials meet the above characteristics perfectly. Combining various materials of different optical properties with biosensors is an effective method to develop novel sensors. Nanoscale proximity enables optically active nanomaterials to be exposed to near- and far-field interactions and participate in energy transfer, plasma interactions and other types of optical processes. The existence of these materials provides new opportunities for energy harvesting, light detection and nanomedicine cognition [8,9,10]. Nanomaterials provide better environmental stability for the sensing platform and increase the specific surface area of the sensitive layer, which enables the biosensor to obtain higher sensitivity and lower detection limit. In addition, biosensors can take advantage of the inherent properties of noble metal nanoparticles to improve the detection performance. For example, surface plasmon resonance (SPR)/localized surface plasmon resonance (LSPR) can be used to improve the electric field [11]. In this way, the sensor precision, signal to noise ratio and other parameters can be improved.

**Figure 1:**
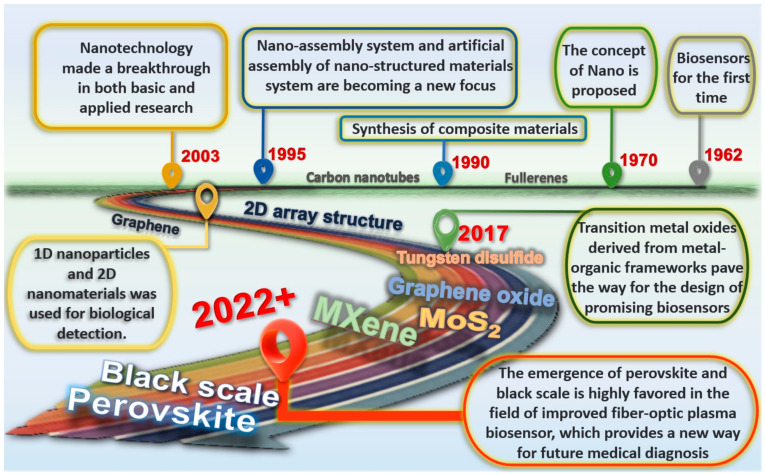
Roadmap to showcase the growth of optically active nanomaterials for biosensing applications.

Reliable and accurate fabricating methods are essential to the functionality of optically active nanomaterials-based biosensors. The roadmap to showcase the growth of optically active nanomaterials for biosensing applications is shown in Figure 1. The methods of nanoparticles synthesis can be divided into “top-down” and “bottom-up” methods. Top-down methods, such as, single- or sub-monolayer nanomaterials can be obtained by breaking the weak van der Waals forces between layers by mechanical stripping methods [12]. In bottom-up methods, such as vapor deposition, hydrothermal solvothermal and solution synthesis, atoms or molecules are assembled step by step into nanoparticles [13,14].

The advantage is that the synthetic homogenous nanostructure can perfectly present the crystal structure.

At present, a variety of biosensing sensing methods have been combined with optically active nanomaterials to test biological substances, such as electrochemical biosensors, fluorescent biosensors, Raman scattering biosensors and SPR biosensors [15]. Bollella et al. gave an overview of the application of graphene and graphene-like materials in electrochemical biosensors. The electrochemical biosensors of two-dimensional (2D) nanomaterials such as boron nitride (BN), graphite carbon nitride (g-C_3_N_4_), transition metal dichalcogenides (TMDs) and graphene were studied. The electrochemical properties of newly modified 2D nanomaterials were summarized in the review [16]. Zhang et al. made a comprehensive overview of electrochemical biosensors based on Metal–organic frameworks (MOFs) and summarized the different strategies of electrochemical biosensors based on different types of MOFs nanocomposites [17]. Ackermann et al. provide an overview of advances in multi-walled carbon nanotubes (MWCNT)-based fluorescent molecular sensors and put forward chemical design strategies for diverse analytes. The insights of fluorescence sensor in the field of photo-physics and molecular recognition were also presented [18]. Aldewachi et al. discussed the strategies, mechanisms and perspectives for the employ of AuNPs as colorimetric biosensors. The interactions between AuNPs and various substrates in the biosensors were analyzed in detail [19]. Recent developments in silicon functional nanomaterials biosensors, bioimaging and cancer therapy were highlighted by Peng et al. The application of silicon nanomaterials in the development of high-performance biosensors and their potential applications in the detection of hypersensitivity and selectivity were discussed, that provides a broad prospect for the detection of biological species such as DNA and protein [20]. Liang et al. reported that a rapidly developing area for surface-enhanced Raman scattering (SERS) biosensor by the use of carbon-based nanomaterials (CBNs) as substrate materials, such as zero-dimensional carbon quantum dots (CQDs), one-dimensional carbon nanotubes (CNTs) and carbon-based core-shells and other nanostructures. Researchers discusses recent research progress of SERS biosensors, especially carbon-based SERS biosensors, for the measure of bioactive molecules was reviewed [21]. Loiseau et al. analyzed various coating strategies (organic, polymer and inorganic) and the effects on the silver nanoparticles (AgNPs) properties of the coatings. The combination of gold and silver for plasma biosensing was investigated [22]. However, all the reviews published so far focus on one or a certain type of optically active nanomaterials, and there is no comprehensive summary of the current optically active nanomaterials and their applications in the field of biosensing.

This review provides a comprehensive summary of various optically active nanomaterials, including carbon-based nanomaterials, inorganic-based nanomaterials, organic-based nanomaterials and composite-based nanomaterials. The applications of biosensors based on optically active nanomaterials are also summarized. Our work provides a platform for understanding nanomaterials and designing high-performance biosensors. Finally, the challenges, and future prospects of optically active nanomaterials biosensors is discussed.

## 2. Optically Active Nanomaterials and Its Types: Based on Their Chemical Compositions

**Figure 2:**
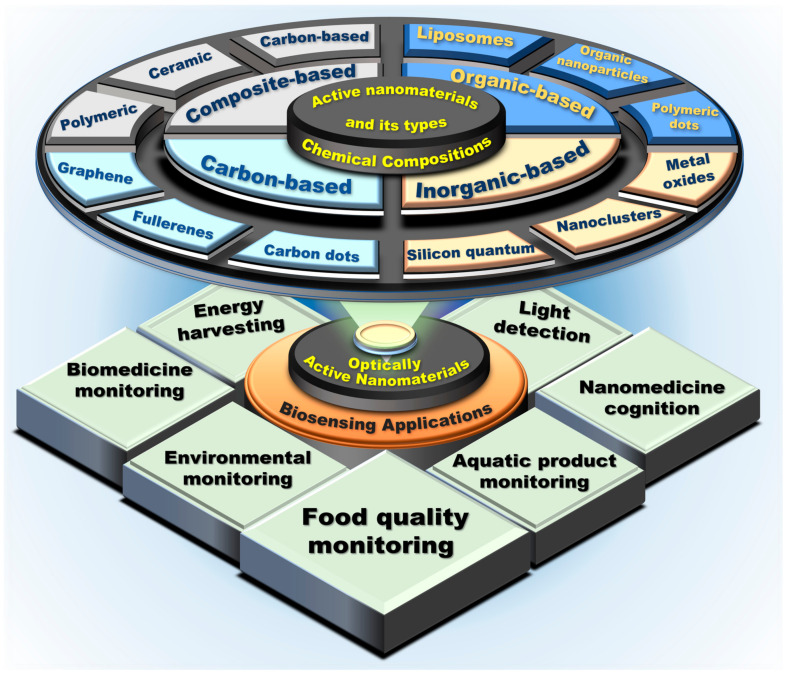
Schematic of optically active nanomaterials-based sensing applications.

Numerous optically active nanoparticles have been utilized for biosensing, primarily carbon-based nanomaterials, inorganic-based nanomaterials, organic-based nanomaterials, and composite-based nanomaterials. Nanomaterials have several uses in various industries, including energy harvesting, biomedicine monitoring, environmental monitoring, light detection, nanomedicine cognition, aquatic product monitoring, food quality monitoring, and healthcare biosensing. Schematic of optically active nanomaterials-based sensing applications is shown in Figure 2.

### 2.1. Carbon-Based Nanomaterials

Carbon-based nanomaterials (CBNs) are gaining popularity as nanomaterials in the fields of science and technology [23]. Numerous carbon allotropes exist, ranging from well-known allotropes like amorphous carbon, graphite, and diamond to recently discovered allotropes like CNT, graphene oxide (GO), graphene quantum dots (GQD), and fullerenes [24,25]. As a result, CBNs, which have recently become valuable and are used in biomedical applications, have drawn a lot of attention for their distinctive chemical. CBNs including CNT, GO, and quantum dots (QDs), have been thoroughly researched for biological applications because to their inherent features. This overview examines current research on CBNs that are used in many medical applications, including cancer treatment, medication transport, and biosensing.

GO is capable of delivering particular responses to the target molecule and engaging dynamically with the probe [26,27]. Fluorescence, Raman scattering, and electrochemical reaction enable the transduction process. On account of this, GO has seen extensive application as a biosensor. The superb physicochemical features of graphene, the strong ionic contact between the positively charged nuclear base and the negatively charged -COOH group, and the sturdy—stacking between the nuclear base and the honeycomb carbon framework are all responsible. Yang et al. developed a glucose biosensor probe based on LSPR with a sensitivity of 0.93 nm/mM for glucose detection at concentrations between 0–10 mM. Then, Yang et al. modified the earlier LSPR-based sensor probe with GO to increase the biosensor’s sensitivity [28,29]. Thereafter, a fiber optic biosensor based on LSPR technology was proposed by Li et al. [11] with the help of functionalized GO, gold nanoparticles (AuNPs), molybdenum disulfide nanoparticles (MoS_2_-NPs), and keratinase (CA) enzyme. 2D materials were widely utilized during the development of biosensors to increase biocompatibility [30,31]. When the concentration of creatinine in a sample increase, the peak wavelength of the detection spectrum shifts to a higher wavelength. It demonstrates the binding of the creatinine solution sample to the enzyme on the probe surface of the sensor that causes a wavelength change in the detecting spectrum. The creatinine sensor has a sensitivity of 0.0025 nm/M, and a LoD of 128.4 μM across a linear detection range of 0–2000 nM [11].

**Figure 3:**
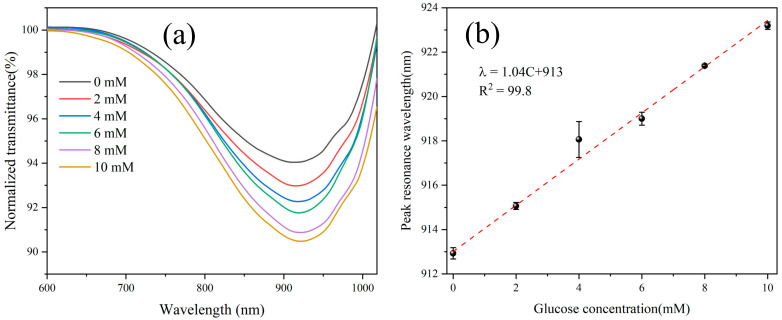
LSPR sensing results of glucose solutions, (**a**) the normalized transmittance, and (**b**) the linearity plot of the sensor. Reprinted with permission from *IEEE Sensors Journal*, Copyright 2022, IEEE [33].

Thereafter, as a result of remarkable structural, mechanical, electrical, and optical properties, CNT is acknowledged as a new generation of nanoprobes. Because of their great chemical stability and sensitivity, high conductivity, high aspect ratio, and quick electron transfer rates, they are ideal for biosensing applications [32]. The immobilization of biomolecules on their surfaces, which improves the identification and signal transduction processes, is a critical component of CNT-based biosensors. These biosensors are fundamentally classified into optical and electrochemical CNT-based biosensors depending on their target identification and transduction techniques. For the fabrication of LSPR-based sensors to obtain reliable detection of human glucose levels, Li et al. [33] developed a special serial four-tapered structure employing multi-walled carbon nanotubes (MWCNTs) and GO. GO and MWCNTs were used to increase the enzyme binding sites, which improved the probe’s sensing capabilities. Glucose oxidase (GOx) enzyme functionalizes the fiber optic sensing probe surface to increase selectivity. The experimental outcomes are shown in Figure 3a. Since the resonance conditions of LSPR heavily depend on the dielectric properties of the surrounding medium, the precious metal particles may be used as sensors to detect RIs [34]. Figure 3b shows that the sensitivity and LOD of the functional probe structure in the linear range of 0–10 mM were 1.04 nm/mM and 0.24 mM, respectively. Additionally, the repeatability, stability, and selectivity of optical fiber probes were evaluated with promising results. CQDs-based biosensors have recently been primarily developed for use in clinical analysis and disease detection [35,36,37,38].

Building on the exceptional photoluminescence, electro-chemiluminescence, and electrochemical behavior of GQD, these have been widely used to detect biological macromolecules, such as DNA, RNA, protein, or glucose molecules, with improved selectivity and sensitivity. Ratlam et al. [39] employed cyclic voltammetry (CV) and amperometry to examine the electrochemical activity of the polyaniline (PANi)/CQDs film toward the detection of dopamine (DA) in neutral solution. The developed film showed good sensitivity for DA sensing with a sensitivity of 8.025 nA·cm^−2^·μM^−1^, a linear range of 10–90 μM and LoD of 0.1013 μM. Increasing DA concentrations also reduced the luminescence of PANi/CQDs in phosphate buffer saline (PBS) solution for fluorescent biosensors. The PANi/CQD fluorescence biosensor has a linear range of 0.1–100 μM and a LoD of 0.0801 μM. The developed PANi/CQDs composites might serve as a material alternative for DA biosensor used in real sample analysis. For the purpose of detecting acrylamide (AM) in food products, Wei et al. developed a simple fluorescent sensors are based on single-stranded DNA (ssDNA) and CQDs [40]. The attachment of ssDNA caused a decrease in the fluorescence intensity of CQDs at 445 nm. The degree of fluorescence decrease was less in the presence of AM than it was in the absence of AM because ssDNA was preferentially attached to AM via hydrogen bonding. According to data obtained under idealized circumstances, the sensing method for detecting AM had a low LOD of 241 μM in the standard solution and a linear relationship spanning with a determination coefficient R^2^ = 0.9895. Furthermore, a high recovery rate in bread crust showed the potential for practical uses of technology. Wu et al. were used GQDs and developed a straightforward and sensitive electrochemical cholesterol biosensor with high conductivity [41]. GQDs facilitates electron transport between the enzyme and the electrode more efficiently. The proposed biosensor has an excellent catalytic performance on cholesterol with a linear concentration range of 16 μM–6.186 mM and LOD as low as 5 μM. In addition, the prepared electrochemical biosensor has good stability and anti-interference ability and has practical application value in cholesterol detection.

**Figure 4:**
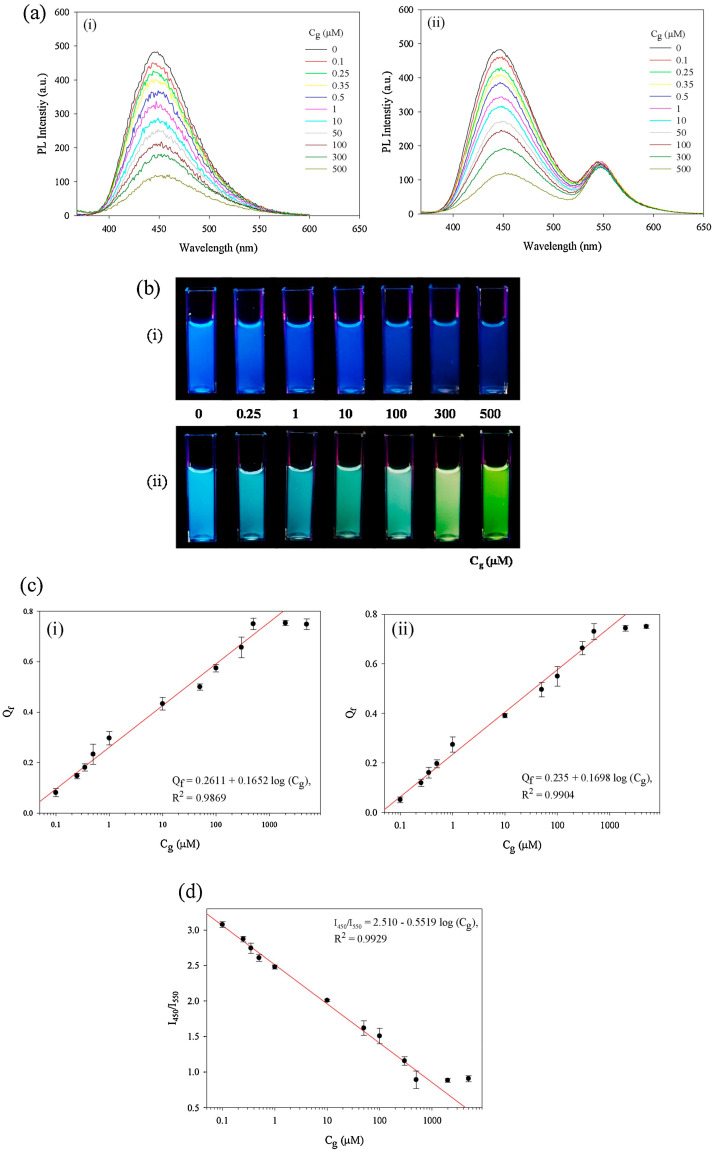
(**a**) PL spectra, (**b**) their fluorescence images, (**c**) Q_f_ (in log scale of C_g_) of a(i) as a function of C_g_ for (i) CD/GOx/HRP and (ii) the CD/Rh6G/GOx/HRP aqueous solutions (0.1 mL) in the vials after glucose aqueous solutions (2.9 mL) were added with different C_g_s, and (**d**) I_450_/I_550_ of the CD/Rh6G/GOx/HRP aqueous solutions as a function of C_g_; numbers in (**a**,**b**) represent C_g_ in μM. Error bars were calculated from measurements from three samples. Reprinted with permission from *Sensors and Actuators B: Chemical*, Copyright 2019, Elsevier [43].

Carbon dots (CD) are an alternative to QDs in biosensing applications due to their low toxicity and biocompatibility. Most sensor platforms with CD are based on biological solution [35,42]. CD aqueous solution is unstable for a long time, and enzymes denature in water when stored for a long time. CD fixation requires reactive groups and reactive CD can be achieved by introducing reactive groups in a bottom-up synthesis process. According to Jin et al., CDs and Rhodamine 6G (Rh6G)-based proportional fluorescent glucose biosensor was developed [43]. Fluorescence quenching by GOx and horseradish peroxidase (HRP) with glucose caused proportionate fluorescence color change in the biosensor. Figure 4a,b shows fluorescence images of vials and photoluminescence (PL) spectra (0.5–500 μM) of CD/GOx/HRP (without Rh6G) and CD/Rh6G/GOx/HRP aqueous solutions. Fluorescence quenching, which lowers monochromatic blue fluorescence, causes the peak intensity at 450 nm in CD/GOx/HRP aqueous solution to diminish. The peak at 450 nm lowers in CD/Rh6G/GOx/HRP aqueous solution as *C* changes. Increasing Cg shows a change from blue to green fluorescence, indicating the presence of glucose in Figure 4b. In Figure 4c, the curves of CD/GOx/HRP and CD/Rh6G/GOx/HRP aqueous solution are shown as functions of Cg, where Cg is the logarithmic scale of C. The linear range is 0.1–500 μM because the quenching of the solution grows linearly until Cg reaches 500 μM. The linear range and LOD for CD/GOx/HRP and CD/Rh6G/GOx/HRP, respectively, are 0.1–500 μM and 0.04 μM as calculated from Figure 4d. Additionally, solid-state glucose biosensor membranes may be utilized as necessary and, unlike aqueous solutions, are naturally stable.

Carbon nanofibers (CNFs) are carbon nanomaterials that have distinctive qualities that make them promising candidates for biosensing applications. These characteristics include a large surface area, biocompatibility, high conductivity, extensive chemical modification, and mechanical stability [44,45]. In the case of biosensors, CNF is not as prevalent as CNTs. Niri et al. developed an electrochemical DNA biosensor based on CNF for hepatitis B virus detection [46].

Due to their high conductivity and surface-to-volume ratio, CNFs are regarded as useful materials for electrochemical biosensors. Electrospun CNF is used directly as the electrode using modified CNF electrode with polymeric glutamic acid (Glu). pDNA was subsequently linked to the Glu-modified CNFs electrode by using CV for detection of target DNA (tDNA). The range of tDNA quantification was 1 pM–1 μM, while the detection limit was 1.58 pM. The electrochemical HBV biosensor has excellent stability, reproducibility, and selectivity, and is able to differentiate complementary DNA from non-complementary and mismatched DNA sequences. Erden et al. developed an ampere-type tyramine biosensor by coupling tyrosinase to a glass carbon electrode modified with CNFs, chitosan, and AuNPs [47]. With the combination of CNFs, AuNPs, and ionic liquids, an accurate, practical, sensitive, and selective method was developed for measuring the tyramine solution. The ideal working range for the sensor is 200 nM–48 μM, sensitivity is 176.6 μA mM^−1^, and LoD is 9.3 nM. The biosensor was tyramine-selective in the presence of other bioamines. Performance study of carbon nanomaterial-based biosensors are well discussed in Table 1.

### 2.2. Inorganic-Based Nanomaterials

Nanomaterials produced from inorganic compounds are chemically stable, extremely adsorptive, biocompatible, and conducive to the immobilization of enzymes or proteins while preserving their biological activity and original structure. It has promising potential applications in chemical and biological sensing. There are numerous methods for producing metal nanoparticles, including grinding and depositing metal nano-islands [60] and electrostatic self-assembly technology for metal nanoparticles [61]. The simplest method is the functional modification or self-assembly of the optical fiber tip. Although the geometric control provided by precision lithography is compromised, the use of this technology allows for the random assembly of sub-types, effectively reducing costs [62]. Therefore, LSPR is a phenomenon in which conducting electrons in metal nanoparticles interact with incident light to produce localized surface plasmas [63]. Due to its high quality and stable properties, AuNPs have the advantages of simple synthesis, excellent chemical stability and good biocompatibility [64]. Their optical properties can also be easily adjusted by their size, shape, and composition [65]. Therefore, AuNP is one of the best choices to excite LSPR and develop plasmonic biosensor [66]. Sharma et al. [67] designed an optical fiber biosensor based on the LSPR phenomenon using AuNPs, that can be used to detect taurine concentration. First, need to grow an amino-silane self-assembly membrane on the fiber probe, and then it is designed to facilitate the attachment of AuNPs, followed by the use of taurine dioxygenase to modify AuNPs to complete the fabrication of the optical fiber sensor. When the enzyme interacts with the reactant, the RI around AuNPs changes, that results in the change of the absorption characteristics of the nanoparticles, that is reflected in the change of the concentration and the change of the absorption wavelength. The optical fiber sensors with sensitivity of 0.0190 AU/mM and detection limit of 53 μM were obtained by this method.

**Figure 5:**
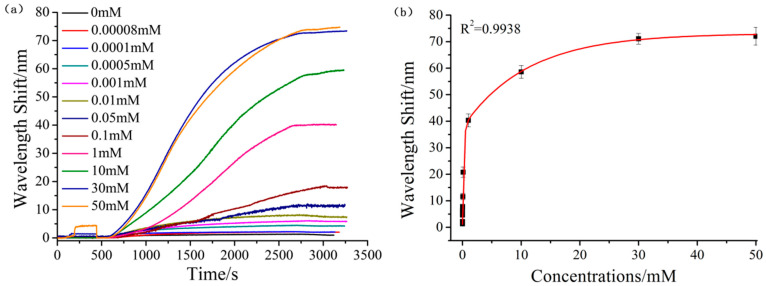
(**a**) SPR sensing at different glucose concentrations and (**b**) linear fitting curve. Reprinted with permission from *Biosensors & Bioelectronics*, Copyright 2018, Elsevier [68].

In addition, the sandwich nanostructures of AuNPs can improve the sensitivity of SPR biosensors through the strong light coupling of incident light at the interface of the nanofilm, so they are widely used as signal enhancement labels with SPR effect. Yuan et al. [68] designed an optical fiber sensor using AuNPs as signal-enhanced tags for glucose concentration detection. First of all, the ion beam sputtering method was used to deposit chromium film and gold film on the optical fiber sensing area. The fiber was then immersed in a mixed solution of 2-aminoethanethiol (AET) and p-mercaptophenylboronic acid (PMBA) with alcohol to functionalize the probe. The fiber structure with Au-film/glucose/AuNPs functionalization was used for the detection of glucose. At this time, the evanescent wave (EW) is strongly coupled to the surface plasmon wave (SPW), and the wavelength shift can be observed. The results are shown in Figure 5a,b. The developed sensor structure has good selectivity and low detection limit for glucose sensing.

**Figure 6:**
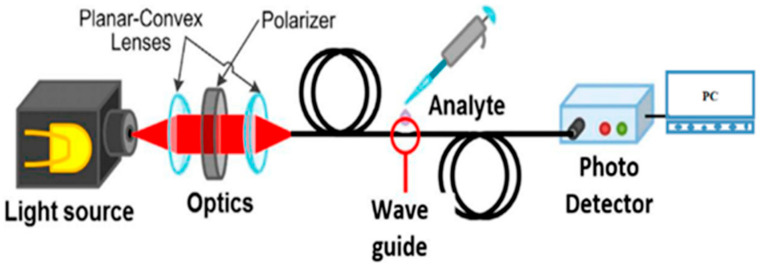
Schematic of the experimental device for ammonium ions detection, adopted from [69].

**Figure 7:**
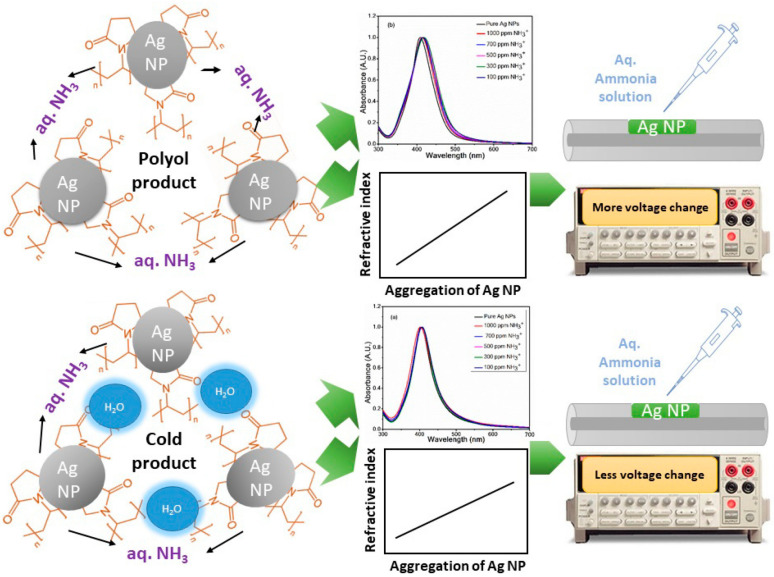
Schematic of the sensing principle for detection of ammonium ions., adopted from [69].

If AgNPs are used, the cost will be further reduced, and this property has a good application prospect in disposable equipment. Potadr et al. [69] investigated the water-phase ammonium ion sensing properties of Polyvinylpyrrolidone (PVA) AgNPs coated optical fiber sensors. PVP-coated AgNPs were synthesized using cold and modified polyol fabrication methods, and then coated on the optical fiber sensing region to detect ammonium ions in water using LSPR effect. The schematic of the experimental setup and sensing principle for detection of ammonium are shown in Figure 6 and Figure 7. The PVP-coated AgNPs have good selectivity for ammonium ion.

Although gold and silver are commonly used to excite SPW as free-electron noble metal materials. However, they are extremely expensive to develop as compared to some metal oxides. The study shows that copper can also be used for SPWs excitation because it has high electrical and thermal conductivity and is a viable alternative to gold and silver. But copper and silver have a similar problem, that is, the metal coating is easy to oxidize. To solve this problem, oxide coating was introduced because of its better chemical stability in the open environment. It has been proved that the fiber-optic RI sensor with high sensitivity can be developed by choosing the suitable oxide coating [70].

In recent years, semiconductor QDs have witnessed a lot of attention due to their strong absorption, high stability and size-adjustable PL properties [71]. Different processes can be used to modify the surface of the QDs, allowing the experiment to obtain the QDs required [72].

**Figure 8:**
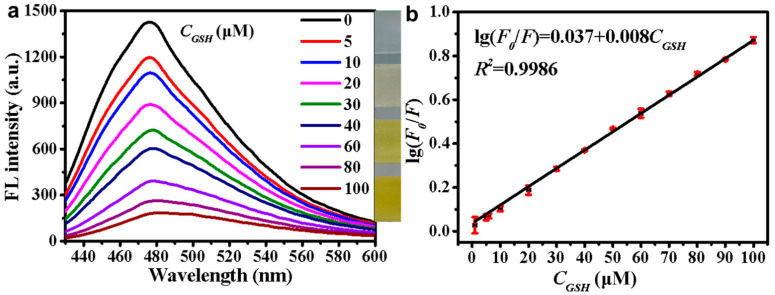
(**a**) Fluorescence spectra of different mercaptan concentrations (the insert has shown that the color gradually changes from colorless to yellow as the concentration of mercaptan increases) and (**b**) log (F_0_/F) as a function of thiol concentrations. Reprinted with permission from *Spectrochimica Acta Part A: Molecular and Biomolecular Spectroscopy*, Copyright 2020, Elsevier [74].

Silicon quantum dots (SiQDs) are semiconductor QDs because of their physical chemistry properties, such as photostability, biocompatibility, water solubility and PL property. It has irreplaceable application value in the fields of biosensor, catalysis and bioimaging [73]. Moreover, the synthesis of SiQDs is simple, hence it is widely used in the field of biosensing. Huang et al. [74] utilized SiQDs developed a fluorescence-based biosensor to detect thiols in living cells. In the experiment, SiQDs were synthesized by one-step synthesis method, and thiols were detected using the internal filtration quenching method of SiQDs. Because SiQDs have a great number of amino groups on their surface, a stable mixture can form between the device and 5,5′-Dithiobis-(2-nitrobenzoic acid) (DTNB). In addition, SiQDs large specific surface area increases the contact area with the object to be measured, that can undoubtedly further improve the sensitivity of the sensor. The linear detection range and LOD of the sensor are 3–100 μM and 0.80–0.96 μM, respectively. The fluorescence spectral intensities and log(F_0_/F) of different mercaptan concentrations are shown in Figure 8a,b, respectively.

**Figure 9:**
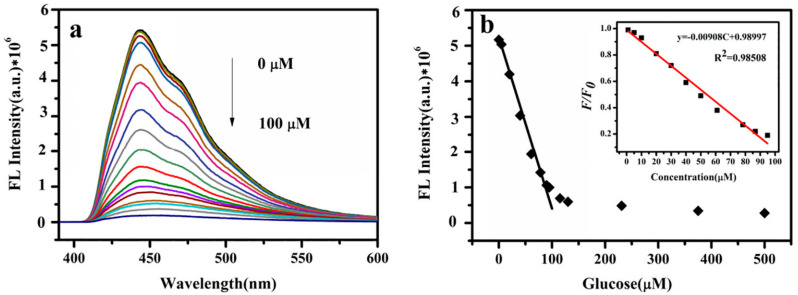
(**a**) Fluorescence spectra at different glucose concentrations and (**b**) linear fitting curve under different glucose concentration. Reprinted with permission from *Sensors and Actuators B: Chemical*, Copyright 2019, Elsevier [75].

Glucose is a monosaccharide in nature, that plays an indispensable role in human metabolism. In the human body, high levels of glucose increase insulin release, that can lead to obesity and diabetes, while low levels can cause hypoglycemia and insulin shock. Therefore, it is necessary to obtain the concentration of glucose in serum in time and effectively. Du et al. [75] realized an enzyme-free glucose sensor using SiQDs. Due to their high biocompatibility and optical characteristics, SiQDs have become one of the most prevalent nanomaterials for biomedical applications. Amino-functionalized SiQDs (NH_2_@SiQDs) are water soluble, have a high fluorescence quantum yield, and are optically stable. Based on the fluorescence quenching of glucose by NH_2_@SiQDs, a glucose nonenzymatic biosensor was developed. The linear fitting curve of fluorescence spectra at different glucose concentrations were shown in Figure 9a,b. The developed blood glucose sensor has been extensively applied to human serum glucose detection.

Liu et al. [73] synthesized SiQDs by hydrothermal method and immobilized them on AuNPs to achieve a composite nanomaterial. They used this nanomaterial to develop a fluorescence-based cysteine sensor with excellent sensing performance.

**Figure 10:**
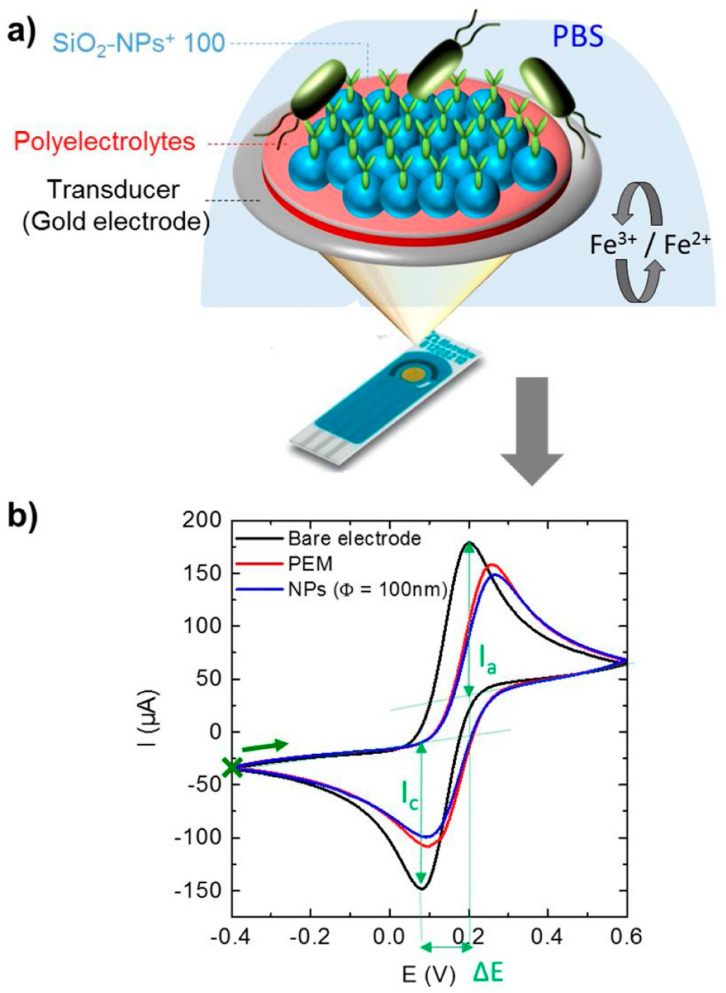
(**a**) Schematic of sensing device for detection of *E. coli* concentration and (**b**) experimental results obtained from cyclic voltammetry. Reprinted with permission from *Spectrochimica Acta Part A: Molecular and Biomolecular Spectroscopy*, Copyright 2019, Elsevier [77].

In addition to providing a biocompatible environment for enzymes, silica nanoparticles (SiO_2_-NPs) can improve the sensitivity of biosensors by promoting the electrical connection between the electrode surface and biomolecules [76]. Mathelié-Guinlet et al. [77] designed an electrochemical immuno-biosensor based on two-step spin-coating process to immobilize SiO_2_-NPs. This sensor can be measured by CV for continuous and unsaturated detection of *E. coli* within five minutes and up to 10^3^ CFU/mL within 30 min. The schematic and experimental results are shown in Figure 10a,b. It holds great promise for practical applications because the electrode is quite simple to operate, and its structure can be adapted to suit a variety of microorganisms.

The focus of research has been on fluorescent dye-doped SiO_2_-NPs as prospective fluorescent probes in biological systems. This is because they have a high fluorescence intensity, good light stability, biocompatible, and simple to modify on the surface to couple with biological systems. Ananda et al. [78] developed a SiO_2_-NPs-based *E. coli* fluorescence sensor. The spherical SiO_2_-NPs were synthesized in this experiment using the reverse microemulsion method. Fluorescence microscope results showed that the bio-conjugated fluorescent SiO_2_-NPs could improve the detection of *E. coli*.

Recently, inorganic nanomaterials have attracted much attention due to their unique physical and chemical properties. Table 2 provides a summary of contemporary sensors based on various inorganic nanomaterials.

### 2.3. Organic-Based Nanomaterials

In recent years, organic-based nanomaterials have drawn a lot of interest because of their distinctive properties, like chemical, physical, and thermal properties. A large amount of literature indicated that organic-based nanomaterials, such as polymer dots, organic nanoparticles (ONPs) and liposomes, are increasingly used in the quantitative and qualitative detection of biomolecules in the field of biomedicine currently.

Polymer dots (PDs) are among the most competitive nanomaterials in optical sensing, due to their excellent characteristics, such as a sizable absorption cross-section, excellent light stability, high luminescence quantum yield, and biocompatibility. The main component of PDs is a semiconductor polymer with π-electron delocalization backbone. PDs can possess different optical properties by changing their molecular structure. In addition, the energy transfer within the particles is promoted by the efficient exciton migration along the polymer backbone, which lays a solid foundation for the construction of the sensing system. At present, the microemulsion method and nano-precipitation technology are the preparation methods of PDs in the application of biosensors [92]. The prevention of infectious diseases caused by bacterial contamination is a crucial part of the protection of human life and health. Therefore, Jo et al. [93] utilized electrode based on PDs coating to establish a reusable biosensor for determination of gram-negative and gram-positive bacteria. In this experiment, PDs were used to modify the electrode to achieve specific binding or interaction with various compounds outside the membrane of the bacterial cells, that would enhance the specific recognition of the sensor. After the bacteria were captured on the electrode surface, the change of the conductivity of the biosensor indicated the presence of bacteria.

**Figure 11:**
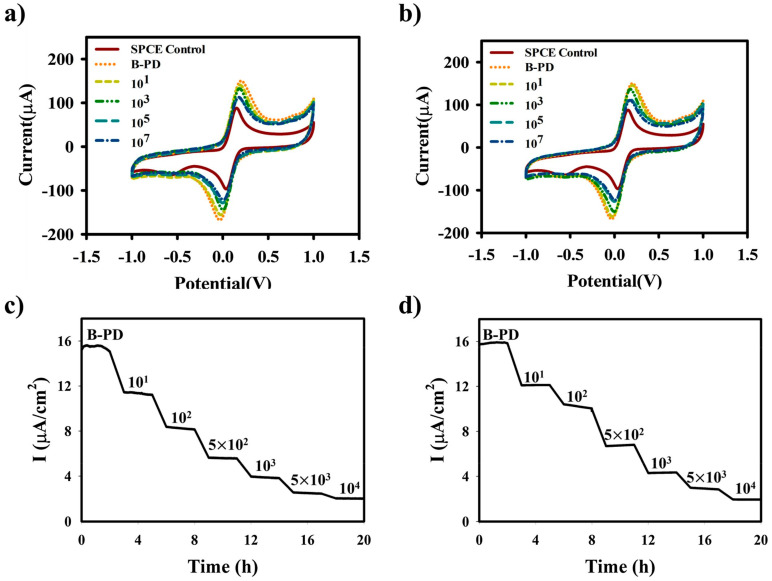
CV measurement results of sensor after determination of (**a**) *E. coli* and (**b**) *S. aureus*. Then, chronoamperometry results of (**c**) *E. coli* and (**d**) *S. aureus*. Reprinted with permission from *Sensors and Actuators B: Chemical*, Copyright 2021, Elsevier [93].

**Figure 12:**
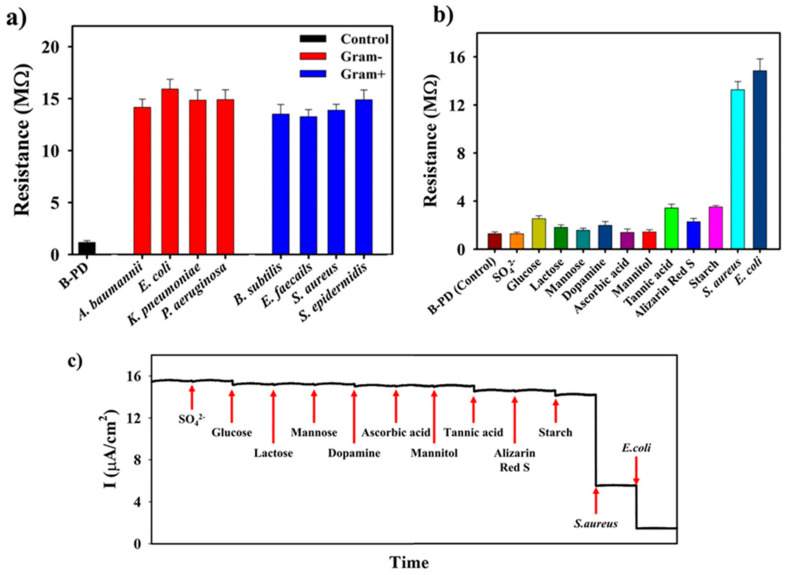
(**a**) The sensitivity of B-PD-coated electrode towards various types of Gram-positive and Gram-negative bacteria using source meter. The selectivity test of B-PD-coated electrode towards various diol compounds and anion (0.1 M) using (**b**) source meter and (**c**) chronoamperometry. Reprinted with permission from *Sensors and Actuators B: Chemical*, Copyright 2021, Elsevier [93].

In this work, *E. coli* and *S. aureus* bacteria can be selectively captured by the PDs-functionalized electrode, and the sensor showed excellent sensitivity to bacteria. Diverse concentrations of *E. coli* and *S. aureus* were detected using CV based on PDs-coated electrodes, as shown in Figure 11a,b, respectively. At the same time, the chronoamperometry was used to detect *E. coli* and *S. aureus*, the results are shown in Figure 11c,d, respectively. The results demonstrated that the limit of detection (LOD) for *E. coli* and *S. aureus* were 10^0.8^ and 10^1^ CFU/mL, respectively. This suggested that the novel biosensor has the potential to detect bacterial contamination, that could help to prevent diseases caused by bacteria. The sensing results of various types of Gram-positive and Gram-negative bacteria are shown in Figure 12a and its various selectivity results are illustrated in Figure 12b,c.

Cancer is an incurable disease that has plagued mankind for many years. Accordingly, it is necessary to design a biosensor to detect cancer cells at present. Won et al. [94] designed a novel electrochemical biosensor based on PDs decorated with MnO_2_. The sensor provided an electrochemical method for detecting cancer cells using the coating surface in addition to realizing the detection of cancer cells based on the fluorescence method. According to the results, the LOD for a biosensor that used an electrochemical method was 3.98 cells/mL, while the LOD for a biosensor that used a fluorescence method was 1995 cells/mL.

**Figure 13:**
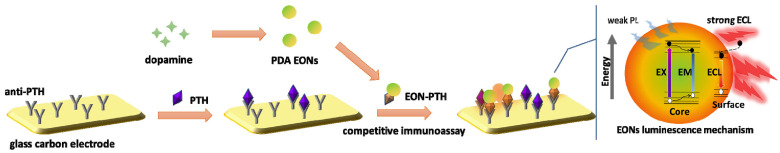
Schematic of ECL-based PTH sensor. Reprinted with permission from *Talanta*, Copyright 2019, Elsevier [96].

**Figure 14:**
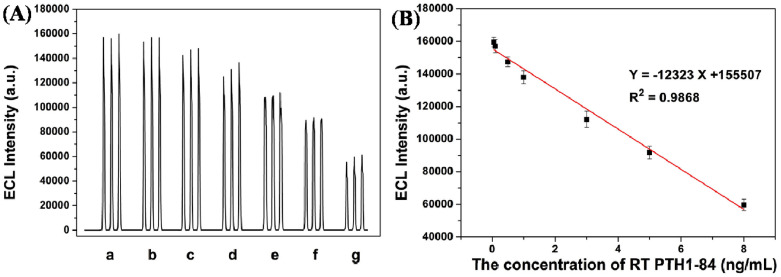
(**A**) ECL traces of different concentrations of PTH (From left to right (a–g): 0.05, 0.1, 0.5, 1.0, 3, 5, 8 ng/mL), (**B**) Calibration curve for quantification of PTH. Reprinted with permission from *Talanta*, Copyright 2019, Elsevier [96].

In the past few years, the use of ONPs to develop chemical and biological sensors has become a trend. ONPs have been widely used in sensing field due to their excellent physical and chemical properties, like strong light confinement, quantum size effect, cost effectiveness and diversity of molecular structure [95]. Li et al. [96] have developed a parathyroid hormone (PTH) sensor based on electrochemiluminescence (ECL) of polydopamine ONPs. The polydopamine ONPs were synthesized by the self-polymerization of dopamine and the oxidation and polymerization processes. In the experiment, dopamine showed excellent physical, chemical properties, and biocompatibility, as well as excellent electrochemiluminescence (ECL) properties. The schematic of the ECL-based PTH sensor is shown in Figure 13. The experimental results suggested that the linear range and LOD of the sensor were 0.05–8 ng/mL and 17 pg/mL, respectively. The ECL intensity of different concentrations of PTH is shown in Figure 14A, and the linear fitting curve in the corresponding linear range is shown in Figure 14B.

**Figure 15:**
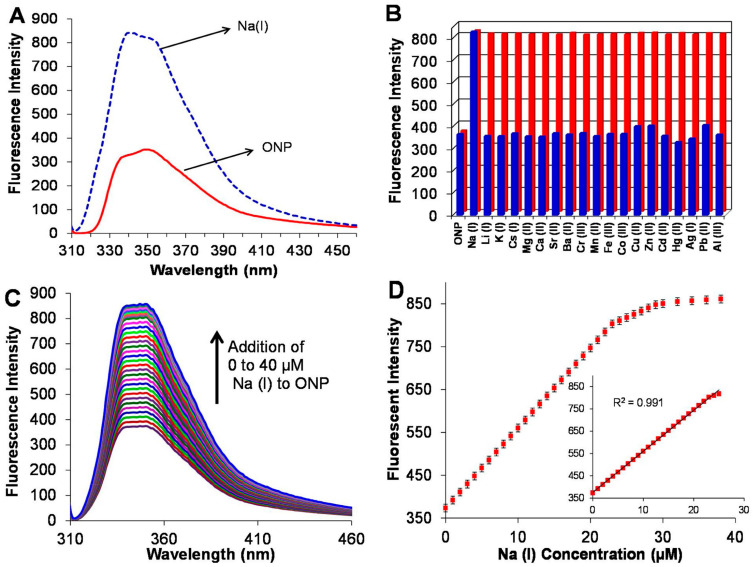
(**A**) Fluorescence emission of ONPs with sodium ions, (**B**) selectivity test of sensors, (**C**) fluorescence emission of ONPs upon titration with sodium ions 0–40 μM, (**D**) relative fluorescence intensity changes with the different concentrations of sodium ions. Reprinted with permission from *Sensors and Actuators B: Chemical*, Copyright 2018, Elsevier [97].

**Figure 16:**
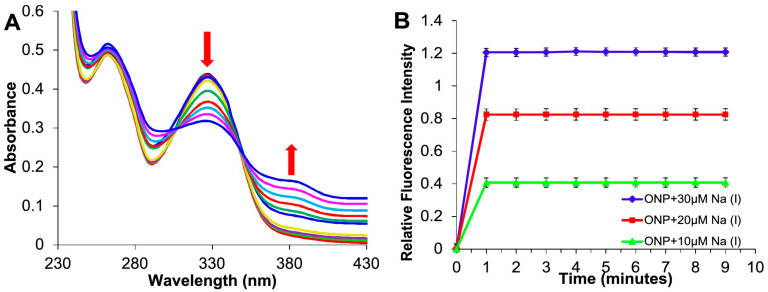
(**A**) Absorbance of ONPs upon titration with sodium ions 0–40 μM, (**B**) the relative fluorescence intensities of three different concentrations of sodium ions varied with time. Reprinted with permission from *Sensors and Actuators B: Chemical*, Copyright 2018, Elsevier [97].

The sodium ion balance in body fluid plays an indispensable role in maintaining normal physiological functions. When serum sodium concentrations exceed a certain threshold, that will increase the risk of stroke, diabetes, kidney disease, and hypernatremia or hyponatremia. Kaur et al. [97] developed a sensor for detecting sodium ions based on Biginelli receptor fluorescent ONPs. ONPs can satisfy the need of measuring the concentration of sodium ion in physiological range. This sensor exhibited sufficient stability in high ion concentrations and in acidic or alkaline environments. Finally, the LOD of the sensor can achieve 22 nM, that indicates the sensor has the potential of practical application. The performance results of the sensors are shown in Figure 15 and Figure 16.

Liposome is a kind of biomimetic material with cell membrane structure, that has great biocompatibility with biomolecules. The membrane property of liposome can be used to modify the sensors, that is more beneficial to the combination of other substances with the sensors [41]. A cholesterol biosensor based on biomimetic liposomes and GQD was developed by Wu et al. [41]. The liposome used in the experiment has a ceramic soft interface and a lipid bilayer membrane, and the electrode surface is immobilized by layer-by-layer self-assembly method, that can serve great biocompatibility and uphold cholesterol oxidase activity. The linear detection range and LOD are 16 µM–6.186 mM and 5 µM, respectively.

**Figure 17:**
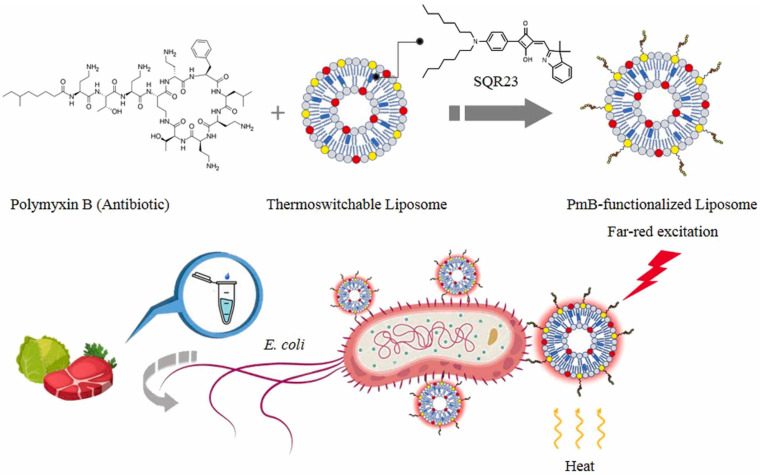
Schematic for quick testing Gram-negative bacteria. Reprinted with permission from *Sensors and Actuators B: Chemical*, Copyright 2022, Elsevier [98].

**Figure 18:**
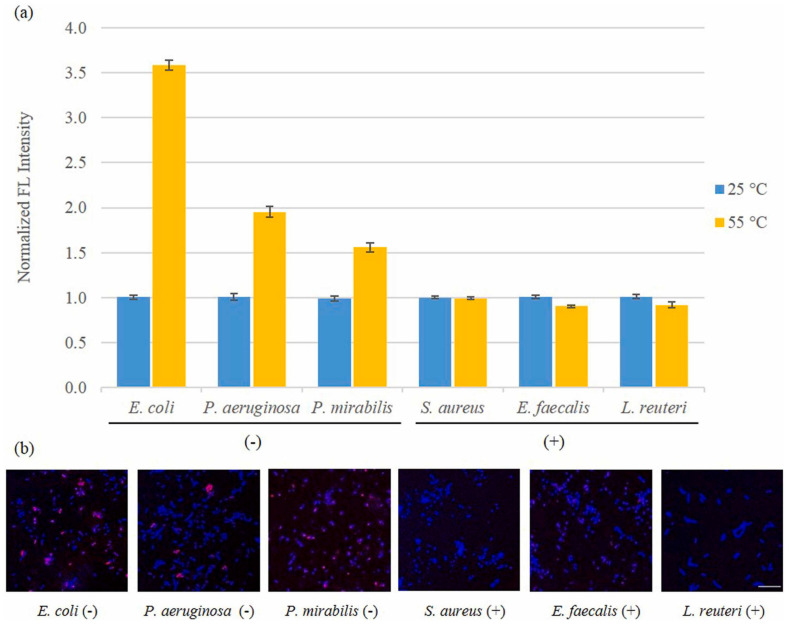
(**a**) Normalized fluorescence intensity of six different Gram bacteria, (**b**) merged confocal images of all six different gram bacteria at 55 °C. Reprinted with permission from *Sensors and Actuators B: Chemical*, Copyright 2022, Elsevier [98].

Tan et al. [98] developed a functional liposome-based assay for Gram-negative. The sensor used polymyxin B as a specific recognition component, that binds to liposomes and utilized fluorescence spectra to distinguish Gram-negative from Gram-positive bacteria and other bacteria. The schematic for quick testing Gram-negative bacteria is shown in Figure 17 and the experimental results are depicted in Figure 18a,b. This opened up a promising method for fast, sensitive, and quantitative detection of Gram-negative using nanoprobes to convert thermal inputs into optical signals.

**Figure 19:**
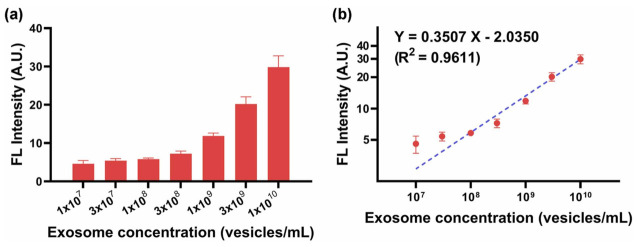
(**a**) Fluorescence intensity according to different concentrations of exosome, (**b**) linear fitting curve between the fluorescence intensity of arrayed PDA vesicles and exosome concentration. Reprinted with permission from *Sensors and Actuators B: Chemical*, Copyright 2022, Elsevier [99].

Exosomes, as a long-distance carrier of intercellular information transfer, contain a variety of biomolecules in the cytoplasm. Therefore, exosomes play an important role in disease diagnosis and in in-vivo drug delivery. Kim et al. [99] developed a multi-target platform-based sensor system with robust selective capture and efficient isolation of exosomes. The sensor is modified by self-organization-based insertion of polyacetylene liposomes. The experimental results show that multi-target platform is more effective than single-target platform for exosome capture. The performance results of the sensor are shown in Figure 19a,b.

Zhou et al. [100] developed a colorimetric biosensor based on a label-free polydiacetylene liposome to detect and recognize different species and levels of bacteria (*P. aeruginosa*, *S. aureus*, *E. coli*, *K. pneumoniae*, *S. pneumoniae*) by simple color changes. Based on the membrane biomimetic cell model and using polydiacetylene liposome molecules to functionalize lipid bilayer, the sensor can detect the colorimetric reaction near the membrane interface. The sensor achieved good sensing performance and provided a new strategy for bacterial species detection and level assessment.

In recent years, organic nanomaterials have been widely used in the field of biosensing due to their excellent optical and biocompatibility properties. In Table 3, the recent development of biosensors based on organic nanomaterials has been summarized.

### 2.4. Composite-Based Nanomaterials

In normal, composite materials refer to the combination of two or more materials with specific chemical and physical methods to form a new type of special materials with the more favorable advantages and characteristics [112]. As for the nano-composite materials employed in the field of biosensing are usually made up of such basic materials like ceramics, carbon-based materials, metals, polymers, etc. By artificial naming for the specific structures to make the distinction, these basic units combined with each other at the molecular level to form a new nanoscale composites-based nanomaterial [113,114]. In common, composite-based nanomaterials are divided into non-polymer and polymer. Here, non-polymer composite-nano-materials are mainly made with inorganic materials, including ceramic-based nanocomposites, and metal-based nanocomposites [115]. In contrast, the constitution of polymer nanocomposites (PNCs) is more complex. Here, only the inclusion of organic or inorganic mixtures in the composition makes the types of PNCs more diverse. Moreover, there are natural materials and, more importantly, synthetic plastics. These materials can be fabricated by industrial processes, which play as the wide range of roles like additives, nano-coated lattices and dispersants, to form a variety of PNCs for biosensing applications [116,117].

**Figure 20:**
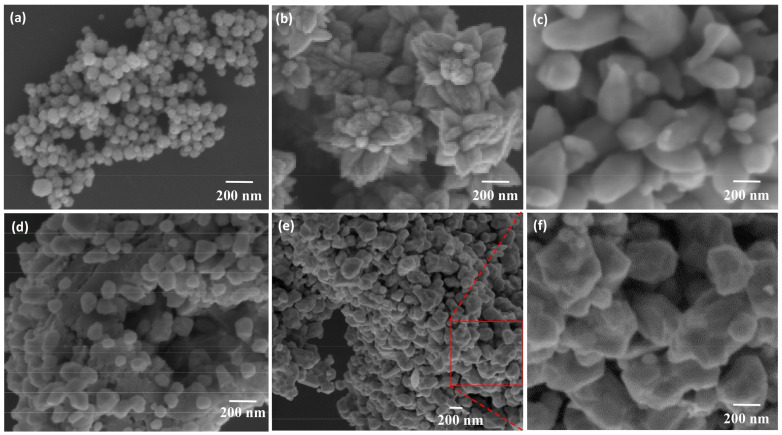
SEM images of microscopic distribution on the sensing region in the synthesis process (**a**) Fe_3_O_4_, (**b**) ZnO, (**c**) ZnO/Fe_3_O_4_, (**d**) Ag/ZnO/Fe_3_O_4_ (**e**) Ag/ZnO/Fe_3_O_4_ (**f**) close up of (**e**). Reprinted with permission from *Analytica Chimica Acta*, Copyright 2019, Elsevier [128].

For the metal-based functional nanocomposites, the matrix phase is mainly composed of containing metals, metallic oxide or alloys [118,119]. One kind of typical material is the zinc-oxide (ZnO)-based composite, for example ZnO/SnO_2_, which has been applied to gas sensor firstly [120,121]. By utilizing N-N heterojunction and synergistic effect between SnO_2_ and ZnO, the composites can form various different morphologies and microstructures such as SnO_2_-core/ZnO-shell nanowires [122], SnO_2_ nanorod arrays/ZnO [123], and SnO_2_-ZnO hetero structure [124]. Those materials have excellent potential for application in biosensing. In their experiment, Liu et al. [125] used ZnO/SnO_2_ composites to show their ability to interact with ethanol gases. The developed sensor system has significantly improved the sensing performance of different concentrations of ethanol gas quantitative analysis. The sensor system can still keep good corresponding results and show excellent stability in up to 200 cycles of repeated experiments [125]. In addition, composite materials based on ZnO and Fe^3+^ are becoming more and more popular, due to which it can form electrostatic repulsive layers with high electron density toward the biological media to eliminate anionic interference. Therefore, the ZnO/Fe_3_O_4_ composites can effectively improve electrode surface modification, that will be widely employed in the research field of electrochemical biosensing [126]. Ghanavati et al. [127] developed a biomolecular detection system for concentration monitoring of naproxen and sumatriptan. It has been reported that they modified the electrode with ZnO/NiO/Fe_3_O_4_/MWCNTs nanocomposite to significantly increase the peak current of electrooxidation. Alula et al. [128] used ZnO/Fe_3_O_4_ composite as an auxiliary material to modify nano-silver for the quantitative detection of uric acid by SERS. The distribution of various surface nanomaterials is shown in Figure 20. The experimental results reported that the proposed composites can participate in the effective interaction between the analyte and the substrate to increase the detection limit. At the same time, the magnetic properties of ZnO/Fe_3_O_4_ composites can greatly simplify the SERS experimental process. In some way, this can effectively reduce the experimental difficulty. Fayemi et al. [129] developed an electrochemical biosensor for the detection of dopamine in real samples. By modifying ZnO/Fe_3_O_4_/NiO nanocomposites on the glassy carbon electrode to improve the electrocatalytic sensitivity of the sensor, they effectively overcome the problems like high ascorbic acid concentration and overlapping voltametric peaks in real samples.

Moreover, due to the excellent electrocatalytic properties of NiO, many interesting metal-based nanocomposites also have many applications in biosensing. For example, Qambrani et al. [130] employed one easy methods called aqueous chemical growth to synthesis the NiO/ZnO nanocomposite successfully, which was used for the electrochemical detection of carbamazepine molecules. The p-n junction can be formed between NiO and ZnO, which greatly improves the electrical properties of the electrode surface, thus effectively improving the sensitivity and stability of the sensor system. It is reported that NiO/MoO_3_ nanocomposites can be synthesized by hydrothermal method as excellent environmental friendly active catalysts [131].

Other metal-based functional nanocomposites like MoS_2_-NPs/CeO_2_-NPs [132], copper oxide-MoS_2_ [133], NiCo_2_O_4_ nanosheets and g-C_3_N_4_ [134], TiO_2_–graphene [135] also used for its excellent biocompatibility, special structural distribution, strong conductivity and large surface area ratio [136].

**Figure 21:**
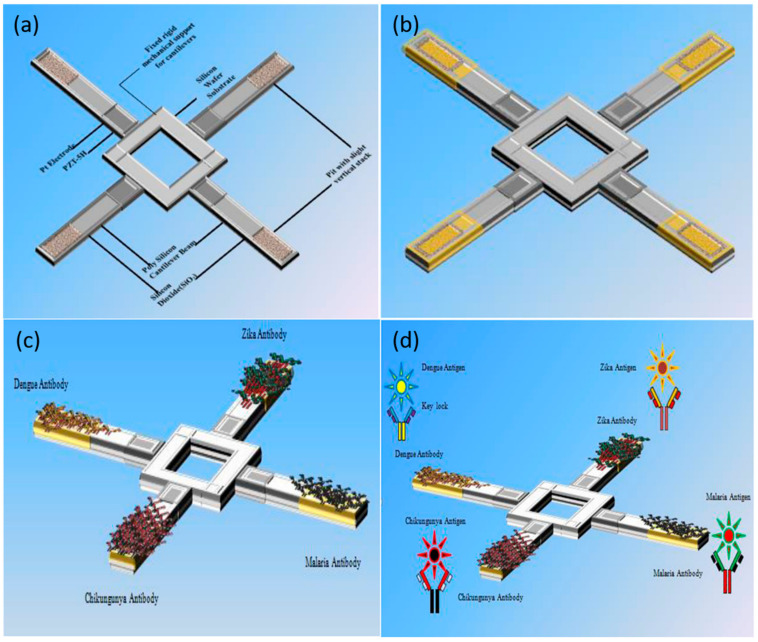
Different coating process results of the designed aster shaped biosensing system based on the array cantilevers, (**a**) represents the various materials used in biosensor design, (**b**) cantilever surfaces after functionalization, (**c**) different sensing layers coated on cantilever surface, (**d**) biosensor exposed to detect specified viruses. Reprinted with permission from Sensing and Bio-Sensing Research, Copyright 2021, Elsevier [140].

The use of ceramics as structural materials is of great significance. Here, the ceramic-based nanocomposites discussed are composed of bulk ceramics and nanofillers, which is effectively dispersed, in order to improve the protection ability, electrical conductivity, optical characteristics and other sensing characteristics of the sensor under extreme conditions. For example, the lead titanate zirconate (PZT), as a kind of inorganic compound, can be used as a high-stability piezoelectric ceramic resonator for a variety of biomolecular detection applications [137]. By controlling the thickness of the ceramic substrate, here the resonant frequency of the resonator will adjust [138]. PZT resonator can be used as an effective alternative to the conventional quartz crystal unit, that provides a new development direction for the construction of sensor arrays in the future. In addition, as an excellent piezoelectric transducer medium, PZT can be also fixed between different silicon layers to achieve the conversion efficiency of voltage spectrum and improve the sensing performance [139]. Figure 21 shows a cantilever array-based biosensor immobilized with PZT and other modified materials [140].

Due to the excellent electrical conductivity and special structural characteristics, carbon ceramic materials are also a major research focus at present, which can be a very promising material in the research field. In common, carbon ceramic materials are usually synthesized by sol-gel synthesis method [141]. By changing the experimental conditions, the pore length, morphology and chemical reaction characteristics of the synthetic material can be adjusted to meet the specific experimental requirements. Due to its large surface area ratio, greater mechanical rigidity, and excellent electrical conductivity, this kind of material can be used as a substrate for enzyme immobilization. In a word, carbon ceramic material is a promising ceramic composite material applied in the biosensing area.

The materials of polymer-derived ceramics (PDCs) are new advanced ceramics produced by preceramic polymers, especially silicon-based precursors. Here, the PDCs can be defined as nanostructured materials with complex nature and unique chemical composition [142]. To prepare such materials, the bulk silicon-based ceramics can be obtained by one-step process directly that solidifying the liquid pre-ceramic polymer through a cross-linking reaction to obtain a mesh hybrid body, and then followed by induced pyrolysis [143].

These materials have been receiving the increasing attention in advanced technology applications with novel combinations of properties. In addition, the silicon-based ceramic composites that frequently employ silicon carbide fiber and carbon fiber as fillers have been shown to greatly increase the structural toughness of the composite layer [144].

Polymersomes, as an interesting artificial polymer vesicle, are sensitive to various external perturbations and widely used in biosensing for the detection of various biomolecules and enzymes. Through the special design and encapsulation of the polymer structure, the functional treatment of the vesicle membrane can be realized. At the same time, material with the excellent biocompatibility and hydrophilicity can be used in fluorescence detection experiments to detect environmental changes. At the same time, its such unique superiorities like unique structure, numerous functional key sites, and strong outer membrane are expected to achieve wider application in biosensing species [145,146]. Researchers used the oxidation properties of disulfide bonds in polymer membranes to achieve quantitative detection of Glutathione molecules at different concentrations in samples [147]. At the same time, researchers have found that by exploiting the difference in reduction potential between tumor and normal tissues, using the vesicles, the targeted delivery of anticancer drugs can be achieved successfully [147]

Zeolite, as an important inorganic material, is also a natural porous crystalline aluminosilicate compound [148]. Zeolite can be used in combination with other organic materials to enhance anionic or cationic functional groups in the polymer matrix. At the same time, with the help of zeolite, the effect of the interaction between the probe and the fixed material on the probe surface can be enhanced to a certain extent. From now, zeolites have been used in different types of sensors, especially in industrial processes like separation, adsorption, catalysis and ion exchange [149,150,151].

Kamaci et al. [152] first proposed a highly sensitive DNA molecular fluorescence biosensor based on poly(azomethine-urethane) (PAMU) and polymer-zeolite.

**Figure 22:**
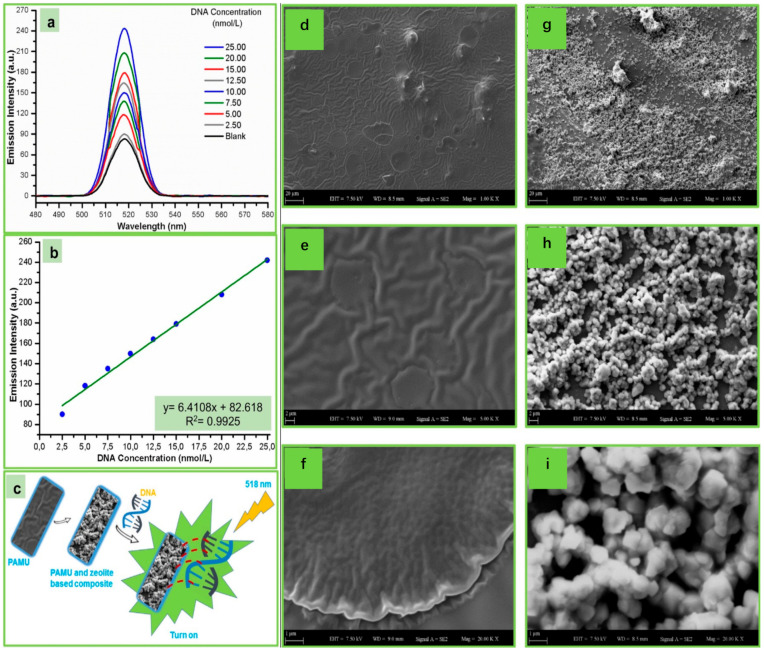
(**a**) Optical spectrum results of zeolite-based composite under the different DNA concentrations conditions, (**b**) linear relationship between DNA concentrations and fluorescence intensity difference above the optimal conditions and (**c**) schematic representation for the proposed biosensor. SEM images of PAMU (**d**–**f**) and zeolite-based composite (**g**–**i**). Reprinted with permission from *Spectrochimica Acta Part A: Molecular and Biomolecular Spectroscopy*, Copyright 2019, Elsevier [152].

The related results and schematic diagram are shown in Figure 22a–c, as well as the SEM images related to the coating composites metals are illustrated in Figure 22d–i. From the results, it can be determined that the proposed sensor exhibited good selectivity and effectively reduces the fluorescence variation. With the use of the novel fluorescent composite containing poly-LRB-azomethine-uret-RPamune) and zeolite, the sensitivity of the pDNAe to DNA molecules was significantly optimized. The linear range of DNA concentration and detection limit are 2.50~25.00 nM/L and 0.095 nM/L, respectively. Compared with inorganic polymer, for example those made of metal or glass, the polymer substrates are seem more flexible and easier to process [153].

Applications of silicon-based nanocomposites with organic or polymer nanophases have gradually expanded to the field of biosensing [154]. This is also due to the fact that polymers with mesoporous silicon dioxide frameworks and polymer nanophases exhibit excellent catalytic properties in the experimental process [155].

**Figure 23:**
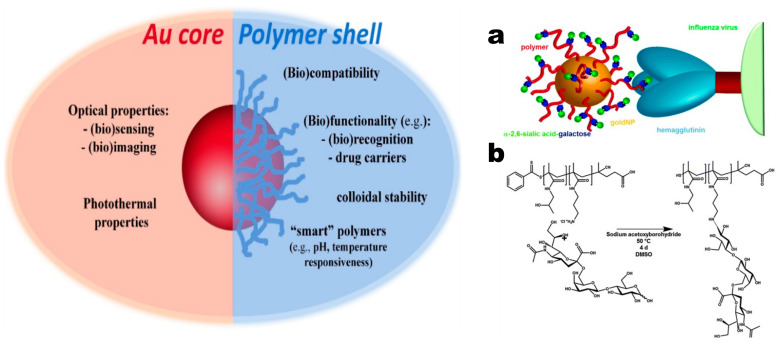
Functionalities associated to polymer/Au nanocomposite particles in some bio applications [156], (**a**) schematic diagram about the recognition process between influenza virus and proposed glyco-goldNP, (**b**) synthesis method about the ligation of α-2,6-sialyllactose to RAFT-based poly(HPMA-*co*-APMA). Reprinted with permission from *Chemical Communications*, Copyright 2016, RSC [157].

In recent years, a new type of Au-based nanocomposites has attracted much attention. This kind of structure is characterized by reversible addition-fragmentation chain transfer (RAFT) polymerization. And as depicted in Figure 23, a polymer shell with a specific functional group is coated on the surface of AuNPs, which have a variety of functions and advantages [156]. This special structure not only enhances bio-recognition and bio-targeting, as shown in Figure 23a, but also provides robustness, stability, functionality, responsiveness, and biocompatibility [157]. However, from the schematic shown in Figure 23b, due to the complexity of its synthesis process, the related sensor research is still in the exploratory stage. It is currently employed for LSPR detection of biomolecular molecules that are used to identify the sugar/lectin system. Increasing the density of specific biological receptors in the polymer shell is a common method for enhancing biomolecular recognition capabilities [158]. Another interesting finding is that stabilization of AuNPs with other glycan copolymers has specific affinity for the protein hemagglutinin on the surface of influenza viruses, which provides new insights into the development of protest vaccines [159].

In addition, several novel nanomaterials, such as magnetic nanocomposites and molecularly imprinted nanocomposites have been utilized extensively in the field of biosensing due to their high selectivity and excellent biocompatibility [160].

## 3. Biosensing Applications

### 3.1. Disease Detection

**Figure 24:**
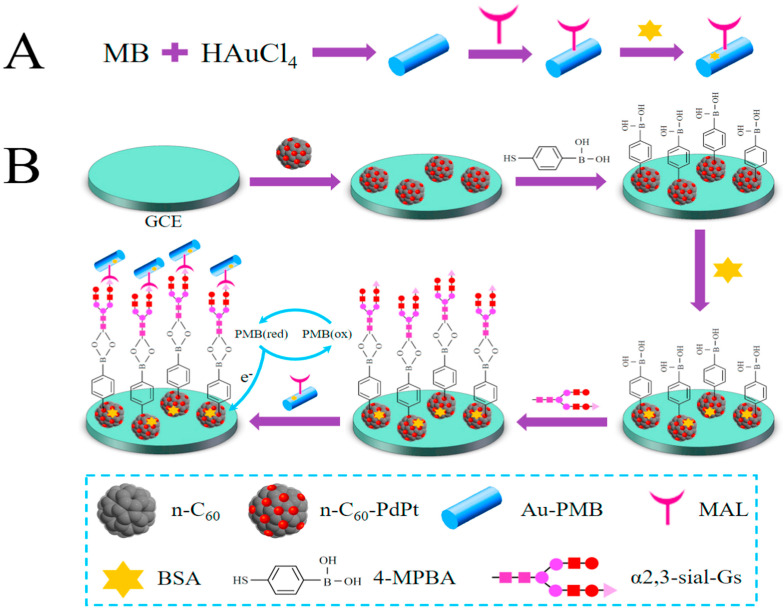
Development of a fullerene–palladium–platinum alloy-based biosensor for the detection of α2,3-sialylated glycans. (**A**) Schematic representation of Au-PMB-MAL, (**B**) Fabrication of the electrochemical biosensor. Reprinted with permission from *Biosensors and Bioelectronics*, Copyright 2018, Elsevier [161].

Fullerenes properties to enhance the electrical conductivity and loading capacity can be easily manipulated by nanocomposite formation in combination with metallic nanoparticles. In this context, Yuan et al. [161] demonstrated the detection of early cancer marker i.e., α2,3-sialylated glycans (α2,3-sial-Gs) by using sandwich-type biosensor consisting fullerenes and metallic nanoparticles composite. The glassy carbon electrode surface has been coated using bimetallic palladium-platinum alloy conjugated to fullerenes functionalized to amino groups (n-C60-PdPt). Nanocomposite has been efficiently functionalized with 4-mercaptophenylboronic acid (4-MPBA) as PdPt can be easily attached to mercapto group. 4-MPBA boron group can be coordinated to the N-acetylneuraminic acid, amide group present in α2,3-sial-Gs structure may be responsible for its detection. Figure 24 shows the schematic representation of proposed sensor.

Differential pulse voltammetry results can be interpreted as final current response of sensor. The proposed sensor exhibits an excellent electron transfer capacity provided by fullerenes and enhanced surface area for in situ palladium-platinum alloy nanocrystals reduction. The detection limit of developed sensor has been recorded to be 3 fg/mL with broad detection range of 10 fg/mL–100 ng/mL. Another study proposed by Singh et al. demonstrated the detection of cancer cells (HepG2, Hepa 1–6, MCF-7, A549) using gold, graphene oxide and copper oxide nanoflowers coated Multi-Core Fiber (MCF) comprising of seven cores arranged in a hexagonal shape spliced with Single-Mode Fiber (SMF) [162]. A developed sensor has been etched in a controlled manner to enhance the evanescent wave (EWs) and coupling of modes between MCF cores and has high refractive index sensitivity. Gold nanoparticles enhance the sensitivity of via LSPR phenomenon, whereas graphen oxide and copper oxide nanoflowers increases the biocompatibility of developed sensor. 2-deoxy-D-glucose coating over fiber has been used as targeting ligand for detection of cancer cells. Proposed sensor exhibited detection limit of 3, 2, 2, 2 cells/mL for HepG2, Hepa1 6, A549, MCF-7 cell lines in linear detection range of 1 × 10^2^−1 × 10^6^ cells/mL.

Glucose plays a major role as energy source in living cells, and its level is clear indication of human health. High blood glucose levels may cause serious health complications and leads to diabetes. Thus, the detection of glucose is an important parameter in treatment and diagnosis of diseases [163]. In this context, owing the property of micron-sized quartz optical fiber like large mode area and enhanced light transmission efficiency, Yu et al. proposed the optical fiber-based glucose biosensor comprised of GOx and CQDs over optical fiber via dip coating method [53]. It mainly works on fluorescent quenching principle of the film on the fiber optic probe and provides fluorescent response curve to glucose concentration. Developed sensor showed the enhanced repeatability and sensitivity for glucose detection, allowing the real time tracking of glucose fluctuation over time period. Fluorescence intensity ration follows the linear range of detection on various ranges and complies well with modified Stern-Volmer equation in range of 10–200 µM/L and 10–100 nM/L with detection limit of 6.43 µM and 25.79 nM, respectively.

Various CNTs-based biosensors have been developed for detection of diabetes mellitus glycemic biomarker. In this context, Hatada et al. [164] proposed the label-free single-wall carbon nanotube-based chemoreceptor-type field effect transistor affinity biosensor for detection of hemoglobin A1c (HbA1c). In this, single wall carbon nanotube acts as transducing element whereas, bacterial periplasmic protein (SocA) acts as receptor. HbA1c proteolytic hydrolysis generates fructosyl valine which can be easily quantified in the detection range of 1.2–1909 nM. Reports also showed that large surface area of CNTs facilitate the efficient functionalization of GOx enzyme and lowers the detection limit to as low as 3 µM [165]. In contrast, reports also showed the elimination of GOx by fabricating MWCNTs scaffolds with cobalt functionalized MoS_2_. Proposed scaffold shows lower detection limit of 80 nM in a linear detection range of 0.2–16.2 mM [166].

Some inorganic nanoparticles, such Fe_3_O_4_, exhibited advantageous properties like magnetic behavior and easy manipulation by external magnetic field, facilitating easy extraction and buffer replacement. In addition to their huge surface area, these materials also offer a good signal-to-noise ratio in biological samples. Lee et al. [167] proposed the iron oxide nanoparticles as core and gold as shell on GSPE for efficient detection of asthma biomarker, eosinophil cationic protein (ECP). The sensitivity of the proposed sensor has been enhanced due to the amplification in electrochemical signal difference. Result revealed that proposed sensor has high sensitivity and can detect in concentration range of 1–1000 nM with low detection limit of 0.30 nM.

Sepsis has been popularly termed as pathogenic bacteria induced systemic inflammatory response syndrome. Now a days, procalcitonin serves as important biomarker in diagnosis of sepsis [168]. For detection of procalcitonin, Chiang et al. developed the immunosorbent assay based on fiber optics nanogold-linked biosensor [169]. Sensor consists of fiber core surface immobilized by capture probe on the surface and AuNPs conjugated detection probe. Presence of analyte and detection probe at biosensor chip facilitate the sandwich-like complex formation consisting of capture probe-analyte-detection probe at fiber core surface via which nano plasmonic absorption of fiber optic EW occur. Authors have functionalized the optical fiber unclad part with antibody against procalcitonin which further conjugated to AuNPs to produce nano plasmonic probes. The sensor exhibits low detection limit of 95 fg/mL (7.3 fM) of procalcitonin with linear response in range of 1 pg/mL–100 ng/mL. Additionally, the sensor exhibited an excellent correlation for sensing procalcitonin in blood plasma and also have several advantages like low-cost instrumentation, and quick response time (~15 min analysis time).

### 3.2. Biomolecules Detection

For biomolecules detection, Sundar et al. [170] proposed the uric acid magneto biosensor comprised of iron oxide nanomaterials. Synthesized nanoparticles have been coated over glassy carbon electrode and thus resultant citric acid-Fe_3_O_4_ nanoparticle modified glassy carbon electrode has been used for uric acid detection via cyclic voltammetry. Oxidation of uric acid occurs at less positive potential of citric acid-Fe_3_O_4_ nanoparticles modified glassy carbon electrode in comparison to naked Fe_3_O_4_ nanoparticles. Proposed sensor exhibits low detection limit of 7.5 µM uric acid and an excellent response in linear range of 7.5 µM–0.18 mM. This enhanced performance of developed biosensor has been attributed due to the large surface area of citric acid-Fe_3_O_4_ nanoparticles which facilitate the constant electron transfer among biomolecules and modified glassy carbon electrode. In another study, Sangubotla et al. [55] proposed the laccase immobilized fiber optic biosensor for sensing of dopamine, biologically significant neurotransmitter based on fluorescent activity. Firstly, bio probe has been constructed with the use of laccase, CD and 3-(aminopropyl)-triethoxysilane and showed quantum yield of 10.2% with significant fluorescence quenching in presence of dopamine at linear range of 0–30 µM and detection limit of 41.2 nM. Further, bio-probe has been immobilized over tapered optical fiber using ethyl cellulose via dip coating method and resultant sensor showed the lowest detection limit of 46.4 nM in a linear concentration range of 0–10 µM. Other than this, developed biosensors showed thermal stability, enhanced photostability and high biocompatibility. Practical application of sensor has been shown in human serum and cerebrospinal fluid.

Further, Baliyan et al. [171] demonstrated the LSPR based senor for detection of triacyl glycerides. The developed sensor has been coated with silver nanoparticles on unclad segment of plastic clad optical fiber and immobilized using lipase enzyme over it. With the change in concentration of triacyl glycerides at sensor probe, peak absorbance wavelength also changes with respect to concentration. Proposed sensor exhibited enhanced sensitivity at 37 °C temperature and 7.4 pH of triacyl glycerides emulsion having 40 s as response time. Sensitivity of sensor has been reported to 28.5 nm/mM of triacyl glycerides solution with detection limit of 0.016 mM. Thus, developed sensor exhibited high stability, selectivity, and reproducibility in real-time sensing and monitoring application. One of the essential components of numerous connective tissues in mammals, Collagen is typically observed as elongated fibrils. It is one of the primary extracellular matrix (ECM) elements that helps cells develop in the majority of tissues [172]. In this context, Kaushik et al. [173] demonstrated the detection of collagen IV using photosensitive (PS) optical fiber-based Mach-Zehnder interferometer (MZI) structure. The developed sensor has been coated with gold nanoparticles, zinc oxide nanoparticles, and polyvinyl alcohol stabilized silver nanoparticles. The MZI is fabricated by sequentially splicing the single mode-multimode-photosensitive-multimode-single mode (SMPMS) fiber segments. Specificity of developed sensor has been facilitated by coating it with collagenase enzyme, and shows the efficient sensing ability in concentration range of 0 ng/mL to 1 µg/mL. It has been observed that the sensing ability of probe has been enhanced by coating of PVA-AgNPs and ZnO-NPs.

### 3.3. Microorganism Detection

**Figure 25:**
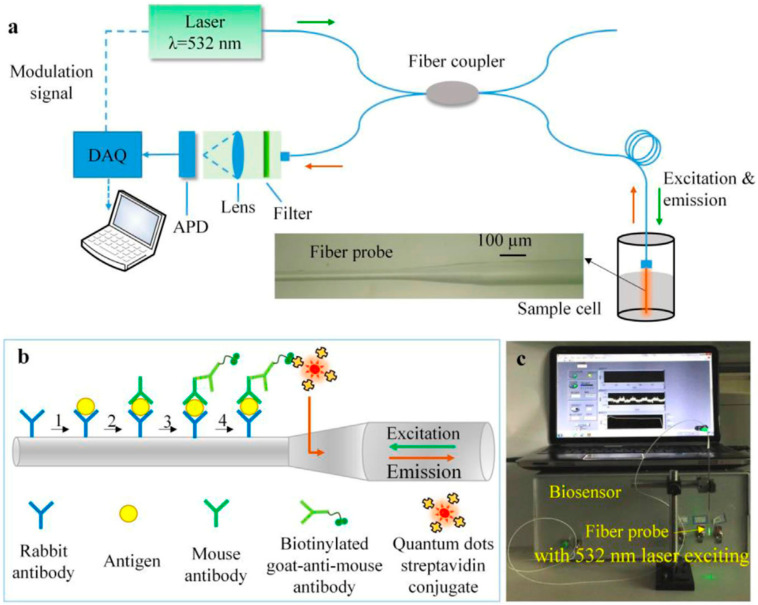
Principle of the fiber optic biosensor, (**a**) schematic diagram of the fiber optic biosensor. (**b**) The principle to identify *S. aureus* using the fiber probe, (**c**) prototype of the fiber optic biosensor, adopted from [72].

Other than biomolecules, nanomaterials can also be used in the detection of cells and microorganism. In this context, Cui et al. [72] developed the optical fiber probe and QDs immunofluorescence-based biosensor for detection of *Staphylococcus aureus* as shown in Figure 25. Developed biosensors exhibit advantage of high specificity between antigen and antibody’s immune reaction, fluorescent labelling, transmission of fluorescent signal along optical fiber as well as high sensitivity and stability of QDs. Detection limit of proposed sensor has been reported to be 1 × 10^3^ CFU/mL within the linear range of 10^3^–10^7^ CFU/mL (correlation coefficient *R*^2^ = 0.9731, *p* = 0.011).

Specificity data revealed that the developed biosensor can effectively distinguish the *S. aureus* from other pathogenic bacteria like *E. coli*, *P. aeruginosa*, *K. pneumoniae* and *A. baumannii*. The whole detection process requires 2 h to complete. Additionally, developed biosensor cannot be affected by the biofilm interference or other secretions as before starting of detection procedure clinical biological specimens needs to be fully liquefied to digest and dissolve the viscous secretions like biofilms. Thus, the developed biosensor can effectively be utilized for rapid and accurate detection of *S. aureus* in real time clinical applications.

In another study, Kaushik et al. proposed the molybdenum disulfide nanosheet functionalized fiber optical immunosensor for effective and sensitive detection of *E. coli*. Monoclonal antibodies against *E. coli* have been immobilized over MoS_2_ functionalized platform via hydrophobic interaction. The detection process requires 15 min and exhibits strong linear relationship (R^2^ = 0.994) among sensor spectral response and bacterial concentration. Specificity and cross reactivity study in presence of other bacterial species i.e., *S. Typhimurium* and *S. aureus* revealed that developed biosensors can be successfully applied for sensing purpose. To establish the real time application, spiked water and orange juice samples have been tested. Result showed the enhanced performance with low detection limit of 94 CFU/mL, high sensitivity of 2.9 nm/1000 CFU/mL and profound specificity in comparison to conventional fiber-based sensor. This sensing technology has promising uses for routinely checking the presence of different harmful bacteria in food and water supplies. Another study by Kaushik et al. proposed the development of optical fiber based immunosensor which consist of two similar chirped LPGs having integrating space of 1 cm which further functionalized with anti-*E. coli* antibodies. The developed immunosensor primarily works on Mach-Zehnder interferometer and detection mechanism based on the shift in unique wavelength by change in RI at IGS region. Present work offers linear detection range of 10–60 CFU/mL with detection limit of 7 CFU/mL [174].

Efficient and early detection of viruses plays crucial role in control of pandemics and epidemics. In this context, Gahlaut et al. [175] demonstrated the surface plasmin resonance based low cost, selective, portable fiber optic sensor for early detection of dengue virus. NS1 protein has been considered as an important biomarker for dengue. Sensors mainly work on the specific binding of antigen-antibody for NS1 antigen detection. Silver-coated unclad fiber has been functionalized with antibody via self-assembled monolayer of alkanethiols and NS1 antigen has been detected in range of 0.2–2.0 µg/mL via wavelength interrogation mode of SPR. Optical fiber has been coated with 40 nm thick silver coating and exhibited maximum resonance wavelength around 500 nm. Sensitivity and low detection limit were found to be 54.7 nm and 0.06 µg/mL, respectively. Detection limit lies in the physiological range of NS1 of inflected blood. Consequently, the current method may offer a very early detection advantage. The setup was successfully tested on actual blood serum samples, demonstrating compatibility with the established techniques. The suggested field-deployable tool has numerous uses in dengue mass surveillance, such as during epidemics and outbreaks. In this context, Kamil et al. also proposed the detection of DENV E protein based on label free tapered optical fiber immunosensor, functionalized with APTES as intermediate linker. Further, Luo et al. [176] demonstrated the immunosensor on the basis of LPG consisting GO for detection of H5N1 virus, common avian influenza virus type. Firstly, GO was deposited over the fiber surface via hydrogen bonding followed by covalent attachment of anti-H5N1 antibodies via amide linkage. Developed immunosensor showed the response time of 10–20 min and total variation of the dual-peak spacing of 10.56 nm. Linear detection range has been reported from 1 ng/mL to 25 µg/mL with low detection limit of 1.7 µg/mL.

## 4. Challenges and Future Prospects

There are numerous future prospects for optically active nanomaterials, particularly in the field of biosensing. Similarly, there are several apt sensors based on nanomaterials that exhibit increased performance but also significant limitations. The vast majority of nanomaterials have a high and nonselective adsorption capability, despite the fact that this topic has not yet been thoroughly researched. Current commercially available nanoparticles include GO, MOF, CNT, g-C_3_N_4_, QDs, and metal nanomaterials. However, its composites are currently undergoing laboratory development, and their repeatability is low. Additionally, the homogeneity of the product is low due to its vulnerability to synthesis conditions such as solvent nature, pH condition, and surrounding temperature. Additionally, nanomaterial-based apt sensors have an effect on our environment. In addition, nanomaterial synthesis methods produce harmful by-products or toxic reactants, necessitating their replacement with green synthesis methods. Some potentially hazardous biosensing reagents produce environmentally harmful byproducts. Nanomaterials are typically discarded after laboratory research that adversely affect the environment, such as the normal life of terrestrial systems and aquatic life. Thus, future researchers must also consider the environmental impact of nanomaterials. There are numerous future prospects for optically active nanomaterials, particularly for the development of compact, portable, and user-friendly in-situ and real-time biosensors. People are developing portable Raman spectrometers, fluorescence detectors, and a miniaturized electrochemical analyzer in order to meet the criteria.

Optical biosensors have evolved in recent years towards high sensitivity, low detection limit, miniaturization, and portability. The physical and chemical features of nanomaterials, such as shape, size, oxidation, and electrical conductivity, have a significant impact on biosensor performance. Nanomaterials’ enormous specific surface area can significantly expand the reaction area of receptors and ligands. With the use of nanomaterials, the performance of biosensors is enhanced. Carbon-based nanomaterials, inorganic-organic nanomaterials, and composite-based nanomaterials are getting increasing interest as nanotechnology advances. The high electrical conductivity of carbon-based nanomaterials, such as CNTs, GO, and CQDs, can unquestionably boost the efficiency of electron transmission, hence enhancing the sensitivity of the sensor. With each passing day, the application potential of organic and inorganic nanomaterials in the sensing sector increases. In the field of biosensing, composite nanomaterials have become increasingly prevalent in recent years.

Nanomaterials have a promising future in the domain of biosensors and have tremendous application potential. Nanomaterials have been widely employed and developed in the fields of biomolecular immobilization, catalytic electrochemical reaction, acceleration of electron transfer, and enhancement of detection sensitivity because of their unique features. The intersection of nanomaterials and biotechnology is the frontier and focal point of international biotechnology research at the present time. Some novel nanomaterials, such as magnetic nanocomposites and molecularly imprinted nanocomposites, will have a vast future in biosensing applications due to their high specificity and superior biocompatibility.

The atomic layers of low-dimensional van der Waals nanomaterials can be altered on the nanoscale scale, hence altering their characteristics and their sensitivity to refractive index. Consequently, low-dimensional van der Waals nanomaterials have garnered the favor of researchers in recent years [177].

Perovskite nanocrystals (PNCs) possess exceptional optical, physical, biochemical, electronics, and optoelectronics properties, features, including narrow emission bands, high quantum yield, and broad absorption spectra [178]. Jia et al. [179] highlighted strategies for promoting the future development of PNCs for chemical/biological sensing applications.

## 5. Conclusions

This article focuses on the study of novel optically active nanomaterials for biosensing applications. On the basis of their chemical compositions, these nanomaterials are categorized as carbon-based nanomaterials, inorganic-based nanomaterials, organic-based nanomaterials, and composite-based nanomaterials. The functionality of optically active nanomaterials-based biosensors is dependent on reliable and precise fabrication procedures. The biosensors based on nanomaterials possess unique chemical and physical properties, such as surface effect, micro size effect, quantum effect, and macro quantum tunneling effect, and exhibit exceptional performance. Thus, these nanomaterials significantly enhance the biosensors’ sensing performance, including response time, sensitivity, detection limit, and specificity. In addition to being extremely specialized for the recognition system, these nanomaterials enable size-dependent signal amplification, charge transfer capabilities, and plasma resonance phenomena. These nanomaterials contribute to the development of useful sensors that will aid in the early identification and treatment of disease, as well as minimize the incidence of illness in humans. Biosensors based on optically active nanomaterials possess the essential properties of portability, compact size, integration, and low cost. Combining diverse materials with varying optical properties with biosensors is an efficient way for developing special sensors.

As nanomaterials, CBNs are gaining popularity in the disciplines of science and technology. Numerous carbon allotropes, including well-known allotropes such as amorphous carbon, graphite, and diamond as well as recently discovered allotropes such as CNT, GO, GQD, and fullerenes, play a vital role in optical fiber optic biosensing. Due to their unique biosensing, CBNs, that have recently become valuable and are used in biomedical applications, have garnered a great deal of attention. Inorganic nanomaterials have stable chemical properties, are extremely adsorptive and biocompatible, and are conducive to the immobilization of enzymes or proteins while preserving their biological activity and distinctive structure. It has promising biosensor and biological detection applications.

Due to their unique chemical, physical, and thermal properties, organic nanomaterials have attracted a great deal of interest in recent years. In the field of biomedicine, polymer dots, organic nanoparticles, and liposomes are increasingly used for the quantitative and qualitative detection of biomolecules. In the area of biosensing, nano-composite materials comprised of ceramics, carbon-based materials, metals, and polymers are also utilized.

Following this, a summary of the uses of biosensors based on optically active nanoparticles is provided. Our work promotes the understanding of nanomaterials and the development of high-performance biosensors. Finally, the challenges and potential future applications of optically active nanomaterial biosensors are highlighted.

## Figures and Tables

**Table 1:** Recently developed carbon nanomaterial-based biosensors.

Mechanism	Materials Used	Analyte	Linear Range	LOD	Ref.
LSPR	GO	Glucose	0–11 mM	n.r. ^a^	[29]
SPR	GO	Glucose	56–390 mM	n.r. ^a^	[48]
SPR	GO	25-hydroxyvitamin D3	2.39 fM–2.4 pM	2.39 fM	[49]
SPR	GO	Glucose	0–6.7 nM	n.r. ^a^	[50]
SPR	GO	Glucose	56–390 mM	n.r. ^a^	[51]
Fluorescence resonance energy transfer (FRET)	GO	Dopamine	0–10 mM	200 μM	[52]
		Nicotine	0–0.7 mM	~10 nM	
		ssDNA	0–0.2 mM	~10 nM	
FRET	CODs	Glucose	10–200 μM	6.43 μM	[53]
			10–100 nM	25.79 nM	
FRET	CODs	Nitric oxide	10–100 nM	9.12 nM	[54]
FRET	CODs	Dopamine	0–10 μM	46.4 nM	[55]
FRET	CODs	Cholesterol	0.01–6 mM	1 μM	[56]
Electrochemistry	CNTs	Uric acid	0.02–2.7 mM	2.8 μM	[57]
SPR	CNTs	Dopamine	0−10 μM	18.9 pM	[58]
Electrochemistry	CNTs	Glucose	0.2–10.6 mM	0.4 μM	[59]

^a^ not reported.

**Table 2:** Comparative study of inorganic nanomaterials-based biosensor.

Mechanism	Materials Used	Analyte	Linear Range	LOD	Ref.
Colorimetric	AuNPs@gelatin	Aflatoxin B1	32.02–448.33 pM	12.81 pM	[79]
Electrochemical	TiO_2_ nanotubes/AgNPs	Heat shock protein 70	1.43 mM–1.43 fM	n.r. ^a^	[80]
Terahertz spectroscopy	AuNPs	MicroRNAs	1 fM–10 pM	14.54 aM	[81]
LSPR	AuNPs	Zearalenone	3.12–149.81 fM	0.32 fM	[82]
Fluorescence	Cu nanocluster	Breast Cancer	500 nM–3 µM	1.7 pM	[83]
Electrochemical	CuONPs/ ZnO nanowires	Glucose	50–500 µM	n.r. ^a^	[84]
Electrochemical	CuO nanoleaves	Glucose	0.005–5.89 mM	12 nM	[85]
Field effect transistors	ZnO nanorods	Serotonin	0.1 fM–1 nM	0.1 fM	[86]
Fluorescence	ZnO-QDs	Cysteine	0.1–600 μM	0.642 μM	[87]
Electrochemical	TiO_2_-NRs/rGO	Dichlorvos	2.26–565 nM	2.23 nM	[88]
Fluorescence	SiQDs	Glucose	1–90 μM	30 μM	[75]
Raman/fluorescence spectroscopic	SiO_2_-NPs	Aflatoxin B1	n.r. ^a^	0.426 aM	[89]
Electrochemical	SiO_2_-NPs	Carcinoembryonic antigen	5 mM–0.8 aM	n.r. ^a^	[90]
Turn-on fluorometric	SiO_2_-NPs	Cephalexin	n.r. ^a^	1.6 μM	[91]

^a^ not reported.

**Table 3:** Comparative study of organic nanomaterials-based biosensors.

Mechanism	Materials Used	Analyte	Linear Range	LOD	Ref.
Fluorescence	NAD(P)H-sensitive Polymer Dot	Phenylalanine	0–2400 μM	3.5 μM	[101]
Electrochemical	Polymer dot	Gram-negative and Gram-positive bacteria	n.r. ^a^	Gram-negative 3.0 CFU/mL and Gram-positive 3.1 CFU/mL	[102]
Electro-chemiluminescence	AIE-Active Polymer Dots	Nucleic acid	n.r. ^a^	32 aM	[103]
Electrochemical	Polymer QDs and C60/MWCNTs- polyethyleneimine nanocomposites	Thrombin	50 fM–20 nM	6 fM	[104]
Electro-chemiluminescence	Carboxyl functionalized polymer dots	Nucleic acid	0.1 fM–100 pM	36 aM	[105]
Fluorescence	Organic nanoparticles	Dopamine	0–10 μM	n.r. ^a^	[106]
Fluorescence	Conjugated polymer nanoparticles	Intracellular telomerase	n.r. ^a^	3 HeLa cells in 400 μL	[107]
Electro-chemiluminescence	Luminol-encapsulated liposome	atxA mRNA	10–300 fM	8.13 fM	[108]
Photo-electrochemical	CRISPR/Cas12 a-mediated liposome	Nucleic acid	0–100 nM	1.6 pM	[109]
Electrochemical	Liposomes	Thrombin	0.1–1000 nM	0.3 pM	[110]
Electrochemical	Biomimetic cerasome/graphene QDs	Cholesterol	16–6186 μM	5 μM	[41]
Colorimetric	polydiacetylene liposome/AuNPs	Thrombin	0–27.03 fM	n.r. ^a^	[111]

^a^ not reported.

## Data Availability

Not applicable.
